# A Case to Overcome the Stigma of Testicular Cancer

**DOI:** 10.7759/cureus.12994

**Published:** 2021-01-29

**Authors:** Maria M Fariduddin, Wajihuddin Syed, Muhammad Naqvi

**Affiliations:** 1 Endocrinology, Diabetes and Metabolism, State University of New York Upstate Medical University, Syracuse, USA; 2 Hematology and Medical Oncology, State University of New York Upstate Medical University, Syracuse, USA

**Keywords:** testicular cancer, missing primary tumor, stigma, cancer screening

## Abstract

Testicular cancer is seen commonly in young males and has a high cure rate if diagnosed and treated early. It clinically presents with painless testicular swelling. We discuss the case of a young previously healthy male with an aggressive testicular cancer which started with a testicular swelling but the primary site underwent necrosis secondary to its own vascular demand thus giving the false impression of resolution, but not before metastasizing to the rest of the body. With this case, we aim to highlight the importance of increasing awareness of testicular cancer and its presenting symptoms in young males and the need to overcome the stigma around the evaluation of testicular swellings.

## Introduction

Testicular cancer is the most common malignancy seen in young males [1]. It generally presents with a painless testicular mass, and infrequently with de novo metastases to the lymph nodes, lungs, abdomen, and bones. We discuss the case of a young male presenting with hemoptysis from an aggressive metastatic testicular cancer without any evidence of a primary mass. 

## Case presentation

**Figure 1:**
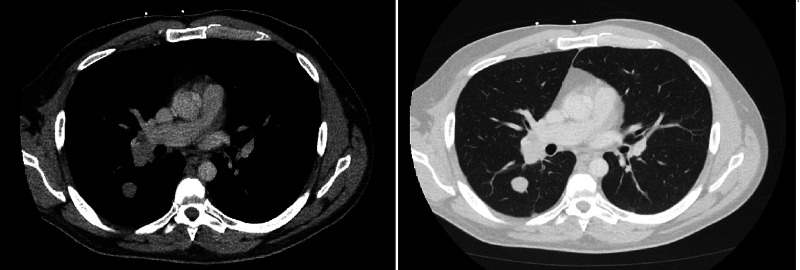
CT scan of the lung with contrast showing right hilar lymph node and right upper lobe mass

**Figure 2:**
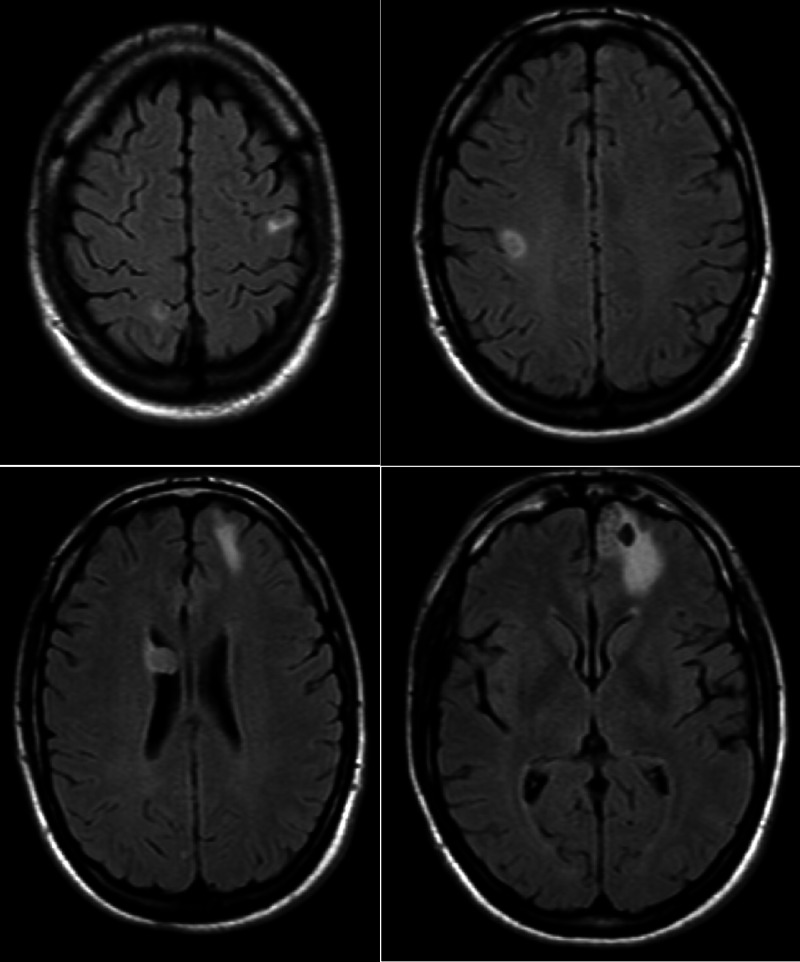
MRI brain with and without contrast showing multiple brain metastases

A 24-year-old male presented to the hospital with multiple episodes of hemoptysis for 24 hours. He had no significant past medical history. CT scan of the chest showed multiple pulmonary nodules with the largest lesions in the right upper lobe of the lung and right hilum (Figure 1). A bronchoscopy and biopsy of the hilar lymph node showed a necrotic focus of metastatic choriocarcinoma. Beta human chorionic gonadotropin (hCG) in the blood was 12,431 mIU/ml (reference range for males <15 mIU/ml). CT scan of the abdomen also showed multiple soft tissue lesions in the mesentery but no testicular or retroperitoneal masses. The primary site of the tumor was elusive; an ultrasound and MRI of the scrotum also did not reveal any masses. Upon further inquiry, the patient mentioned he had noted swelling in his right testicle a few weeks prior to the presentation which he believed was from intense work-out that he is required to do as a part of his job, and did not seek care for it since it was not painful and resolved in a few days on its own. Two days into his admission he developed a severe headache and intractable vomiting; an MRI of the brain showed multiple enhancing metastases throughout the brain (Figure 2). He was started on combination chemotherapy regimen with bleomycin, etoposide, and cisplatin (BEP) and also underwent a short course of whole-brain radiation therapy. With each cycle of chemotherapy, he would have a good response with beta hCG trending down immediately following the chemotherapy, but it would rise in the time his next cycle was due. Considering the laggard response in beta hCG, he also underwent right orchiectomy to possibly eliminate the sanctuary site. Pathology of the right testicle showed a 5 mm focus of fibrosis but did not show any evidence of the tumor. Imaging after four cycles of BEP chemotherapy did show some response, but he continued to have a heavy burden of disease. He was being considered for high-dose chemotherapy with rescue autologous stem cell transplantation, but before this could be set up, his disease progressed rapidly with beta hCG reaching 32,784 mIU/ml. He was started on second-line chemotherapy with paclitaxel, ifosfamide, and cisplatin. Unfortunately, while undergoing this regimen he had a massive pulmonary hemorrhage leading to acute respiratory distress syndrome (ARDS), and eight months after his diagnosis, he died from complications of his disease.

## Discussion

Testicular cancer is the most common form of solid tumor malignancy among young males between 15-35 years of age [1]. The majority of testicular tumors are germ cell tumors and are divided into seminomas and nonseminomas. Testicular germ cell tumors commonly present with a painless mass in one testicle and more than 90% of these are localized to the testicle at diagnosis. Testicular tumors are amongst the tumors with the highest cure rates. Localized tumors have five-year overall survival rates exceeding 99% and even the tumors with the worst prognosis have a five-year overall survival of more than 70% with appropriate treatment [2,3]. Germ cell tumors can be extragonadal if there is no evidence of tumor in the testicles (or ovaries in women) and occur in the mediastinum or retroperitoneum. Mediastinal nonseminomas carry a significantly worse prognosis and present with a mediastinal mass usually in the anterior mediastinum.

The diagnosis of testicular cancer is made with radical orchiectomy which is often curative in early-stage tumors. The patient discussed in this case presented with hemoptysis from pulmonary metastases and had abdominal and brain metastases at diagnosis. The site of origin of his tumor however was elusive as he did not have a primary mass in either of the testicles, mediastinum, or the retroperitoneal abdomen. He had a very aggressive form of choriocarcinoma and with the history of right testicular swelling a few weeks before presentation, it was believed that the primary tumor did originate in the testicle but was “burnt out” because of the high vascular demand in the tumor - as evidenced by necrosis and vascularity in the hilar lymph node - but not before it had already metastasized. This is a very rare presentation of testicular tumors and tends to be seen in aggressive forms of germ cell tumors like choriocarcinoma. In the past century, fewer than 80 such cases with a burnt-out testicular primary tumor have been reported worldwide [4-7].

Risk factors for developing testicular cancer include family history, testicular atrophy, cryptorchidism, and prior history of contralateral testicular cancer. Routine testicular self-examinations have been noted to increase the rate of early detection of testicular cancers in isolated reports, however, there is not enough data to suggest that this improves long term survival [3]. Serum tumor markers such as alpha-fetoprotein (AFP), human chorionic gonadotropin (beta hCG), and lactate dehydrogenase (LDH) are useful for diagnosis and surveillance but are not specific or sensitive enough for screening asymptomatic males [8,9]. Patients with a history of cryptorchidism have a much higher risk of developing testicular cancer in their lifetime and monthly self-examinations and yearly clinical examinations are recommended in patients with this specific history [10,11].

As of this publication, there are no routine screening modalities recommended for the detection of testicular cancers in the general population, and the diagnosis is made when patients present with clinical symptoms [12]. Painless swelling of the testicle, testicular firmness, scrotal heaviness, and gynecomastia are among the early signs of testicular cancer. There is a significant stigma associated with testicular swelling and young patients are hesitant to seek care for testicular problems. A survey done by Cleveland Clinic revealed close to 40% of the patients presenting with testicular swellings waited to see their doctors until they felt the issue was serious or they developed other symptoms [13]. Burnt out testicular primary like this patient’s tumor poses additional challenges in diagnosing these cancers early because the disappearance of the testicular mass gives the patient a false impression of resolution of the problem he might have had. Considering that these tumors occur mostly in young males and have high rates of cure, there is an urgent need for increasing awareness of presenting signs of testicular cancers in this age group to overcome the stigma of testicular swellings and encourage evaluation to prevent delay in the diagnosis and treatment of testicular cancers at an early stage.

## Conclusions

This case highlights the importance of increasing awareness of early signs of testicular cancer in young males to encourage evaluation of testicular swellings as well as education among clinicians to recognize the stigma behind reporting these in their young male patients. The patient discussed in this case had a swelling in his right testicle but because it was resolved, he did not have it evaluated. This stigma associated with the masculine culture around young men provides a major barrier in improving awareness of the risks involved with ignoring painless testicular swellings, even the ones which seem to be self-limiting, albeit falsely, like in this case. Testicular tumors are among the cancers with the highest cure rates if recognized and treated early. Concluding that this patient might have had a different outcome had he got an early evaluation would be speculation since choriocarcinomas tend to be quite aggressive. The lesson we take from this young man’s unfortunate outcome is to educate young male patients and their clinicians to get timely evaluations even for seemingly trivial testicular swellings so that these tumors are detected early and treated appropriately to improve the odds of cure and survival.
